# Supplementary material for: Causal Relationship between Aging and Anorexia Nervosa: A White-Matter-Microstructure-Mediated Mendelian Randomization Analysis
Source: Biomedicines. 2024 Aug 16;12(8):1874. doi: 10.3390/biomedicines12081874 (PMC11351342; doi:10.3390/biomedicines12081874)
Supplement: Supplementary file 1 [file biomedicines-12-01874-s001.zip › Supplementary Figure/Figure S4/Singe SNP plot of MR analysis for TL to ubm-b-1700.pdf]

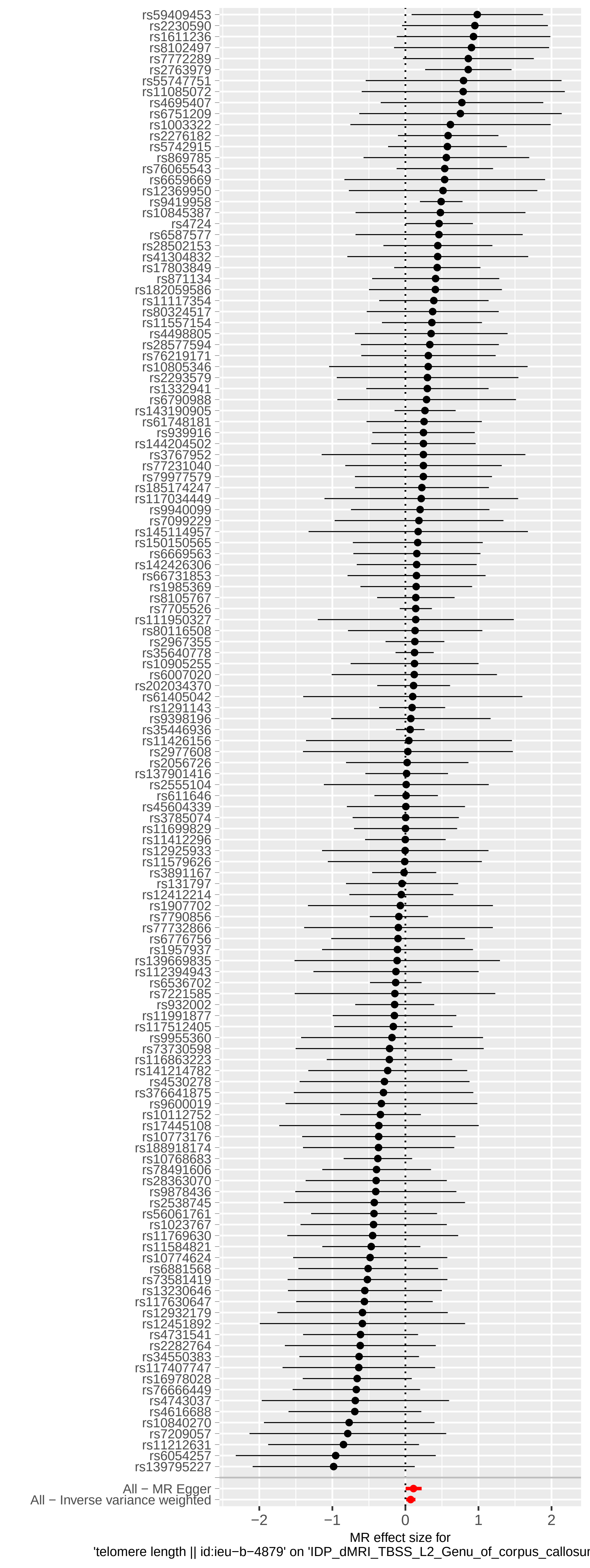
rs59409453
rs2230590
rs1611236
rs8102497
rs7772289
rs2763979
rs55747751
rs11085072
rs4695407
rs6751209
rs1003322
rs2276182
rs5742915
rs869785
rs76065543
rs6659669
rs12369950
rs9419958
rs10845387
rs4724
rs6587577
rs28502153
rs41304832
rs17803849
rs871134
rs182059586
rs11117354
rs80324517
rs11557154
rs4498805
rs28577594
rs76219171
rs10805346
rs2293579
rs1332941
rs6790988
rs143190905
rs61748181
rs939916
rs144204502
rs3767952
rs77231040
rs79977579
rs185174247
rs117034449
rs9940099
rs7099229
rs145114957
rs150150565
rs6669563
rs142426306
rs66731853
rs1985369
rs8105767
rs7705526
rs111950327
rs80116508
rs2967355
rs35640778
rs10905255
rs6007020
rs202034370
rs61405042
rs1291143
rs9398196
rs35446936
rs11426156
rs2977608
rs2056726
rs137901416
rs2555104
rs611646
rs45604339
rs3785074
rs11699829
rs11412296
rs12925933
rs11579626
rs3891167
rs131797
rs12412214
rs1907702
rs7790856
rs77732866
rs6776756
rs1957937
rs139669835
rs112394943
rs6536702
rs7221585
rs932002
rs11991877
rs117512405
rs9955360
rs73730598
rs116863223
rs141214782
rs4530278
rs376641875
rs9600019
rs10112752
rs17445108
rs10773176
rs188918174
rs10768683
rs78491606
rs28363070
rs9878436
rs2538745
rs56061761
rs1023767
rs11769630
rs11584821
rs10774624
rs6881568
rs73581419
rs13230646
rs117630647
rs12932179
rs12451892
rs4731541
rs2282764
rs34550383
rs117407747
rs16978028
rs76666449
rs4743037
rs4616688
rs10840270
rs7209057
rs11212631
rs6054257
rs139795227
All − MR Egger
All − Inverse variance weighted
−2
−1
0
1
2
MR effect size for
'telomere length || id:ieu−b−4879' on 'IDP_dMRI_TBSS_L2_Genu_of_corpus_callosur
